# Formulation and Evaluation of the In Vitro Performance of Topical Dermatological Products Containing Diclofenac Sodium

**DOI:** 10.3390/pharmaceutics14091892

**Published:** 2022-09-07

**Authors:** Mahima Manian, Piyush Jain, Deepal Vora, Ajay K. Banga

**Affiliations:** 1 Center for Drug Delivery Research, Department of Pharmaceutical Sciences, College of Pharmacy, Mercer University, Atlanta, GA 30341, USA; 2 CMC Product Development, Dermavant Sciences, Durham, NC 27560, USA

**Keywords:** topical, semi-solid, in vitro, NSAID, diclofenac

## Abstract

The selection of an appropriate vehicle in a semi-solid topical product is of utmost importance since the vehicle composition and microstructure can potentially cause changes in drug–vehicle or vehicle–skin interactions and affect drug release and subsequent permeation into and across skin. Hence, the aim of this study was to evaluate different semi-solid formulations containing diclofenac sodium for the physicochemical and structural performance of excipients used and various physiological factors governing permeation of drugs applied to skin. The formulations (emulsion, emulgel, gel, and ointment) were prepared using conventional excipients and were found to be homogenous and stable. Rheological analysis demonstrated characteristic shear-thinning and viscoelastic behavior of formulations. The mean release rate of the gel formulation (380.42 ± 3.05 µg/cm^2^/h^0.5^) was statistically higher compared to all other formulations. In vitro permeation using human skin showed a significantly greater extent of drug permeation and retention for the emulgel formulation (23.61 ± 1.03 µg/cm^2^ and 47.95 ± 2.47 µg/cm^2^, respectively). The results demonstrated that the different formulations influenced product performance due to their inherent properties. The findings of this study demonstrated that a comprehensive physicochemical and structural evaluation is required to optimize the in vitro performance for dermatological formulations depending on the intended therapeutic effect.

## 1. Introduction

The largest organ of the integumentary system, the skin, is important for regulating body temperature in humans as well as serving as a barrier against the outside environment. The skin is therefore most vulnerable to environmental and physical stresses. The development of skin diseases and disorders that may be treated with topical formulations may also be influenced by autoimmune disorders, drug-induced hypersensitivity, and other circumstances. The drug can be loaded in various topical formulations to exert its activity on the surface tissue layer, via penetration into the deeper layers of skin to reach the target site, or via systemic delivery, depending on the physicochemical properties of the active ingredient, the targeted site, and formulation strategies used [1]. There is no one vehicle for topical medications for every pharmaceutical active due to the multidimensional complexity of topical products. For optimal therapy, each drug at each concentration needs a unique vehicle (structural matrix or other components) [2]. Simplicity in formulation design, along with an integration of the different components, is desirable. However, topical formulations are complex, and a thorough evaluation is required during formulation development in order to avoid any unwanted interactions and meet patient acceptance. While developing a new topical drug product, a thorough evaluation of the in vitro behavior of the semi-solid dosage form helps to ensure it meets the intended clinical performance. For semi-solid formulations, viscosity is important because it can change the rate at which the drug diffuses from the vehicle, which can affect how the drug is released [3]. Viscosity is seen as an indication of a product’s stability and is related to the robustness of the internal structure. Therefore, it is important to completely understand the rheology of the product since it may determine both the release and permeation of the drug to and across the skin from semi-solid topical dose forms such as creams [4].

Similarly, while developing generic products, the product quality and performance must be comparable to the innovator product in order to ensure therapeutical equivalence. A majority of the drugs applied topically at the site of action require clinical BE endpoint studies, including Nonsteroidal Anti-Inflammatory Drugs (NSAIDs) such as diclofenac sodium [5]. Diclofenac acts by blocking prostaglandin formation when applied topically and may be beneficial for the treatment of acute and chronic pain [6]. Additionally, it has been claimed that diclofenac may work through multiple routes, including altering the synthesis of IL-6, inhibiting the thromboxane pathway, and suppressing NMDA receptor hyperalgesia [7]. The mechanism of action proposed for the therapy of actinic keratoses is the inhibition of cyclooxygenase-2 (COX-2), which leads to a reduction in prostaglandin synthesis and inhibition of cell differentiation and angiogenesis, induction of apoptosis, and changes in cell proliferation [8]. Current marketed topical diclofenac products include over-the-counter Voltaren^®^ (diclofenac sodium topical gel 1%) (GSK Consumer Healthcare, Warren, NJ, USA) and prescription products Pennsaid^®^ (diclofenac sodium topical solution 2%) (Horizon Therapeutics Ireland DAC, Dublin, Ireland) and Solaraze^®^ (diclofenac sodium topical gel 3%) (Fougera Pharmaceuticals Inc., Melville, NY, USA). The bioavailability and clinical efficacy of topically applied diclofenac have been studied extensively [9,10]. In clinical research, it was discovered that 1% diclofenac sodium gel’s systemic exposure was roughly 5–17 times lower than oral diclofenac administration’s minimal systemic exposure [11]. As a result, the ability to avoid first-pass metabolism makes topical delivery of NSAIDs preferable to oral delivery.

The in vitro performance of a topical product is a major component of the Quality Target Product Profile (QTPP) framework [12] and is critical during the development of both innovator and generic dermatological products. Semi-solid dosage forms are known to exhibit intrinsic variability, which establishes the need for demonstrating reasonable equivalence in the case of generic products. In recent years, several articles have been published highlighting the importance of evaluating the ‘In vitro performance’ of topical formulations [3,13,14]. The major formulation goal for a generic topical drug product is quantitative sameness (Q1), qualitative sameness (Q2), and microstructure sameness (Q3) compared to the reference product. A number of Product Specific Guidance documents (PSGs) have been published by the FDA for complex generic drug development based on alternate approaches to establishing bioequivalence [15]. Several PSGs have also been published for topical products in order to demonstrate BE with the reference product (Acyclovir cream, Clindamycin gel). However, these non-clinical, surrogate methods often show variable results due to the extremely complex nature of the product and the interdependent relationships between the structural properties, manufacturing process, performance, etc. The therapeutic efficacy of any topical formulation is dependent on the vehicle, which can potentially influence the drug release from the formulation and subsequent skin permeability. In addition, the structural behavior of the product may potentially exhibit a synergistic effect on the release and permeation behavior of the formulation. Hence, the aim of this study was to evaluate the extent to which the selection of a suitable topical vehicle affects both the in vitro performance and profile of a widely available model NSAID such as diclofenac sodium. The rheological evaluation of the various semi-solid formulations was carried out for the goal of this study in order to offer insightful information on the structure and viscoelastic qualities that have been shown to affect drug release and diffusion [16]. However, in research and development, it can be used to assess the vehicle design, performance, and how quickly the drug may be released from the formulation into the skin. Historically, in vitro release testing (IVRT) has been frequently employed to ensure product sameness. Consequently, the test may be used to determine how well a topical treatment performs in vivo [17]. The impact of the various vehicles on the in vitro release of diclofenac sodium was assessed using an approved in vitro release testing method. In addition, skin permeation studies were also performed in order to determine if there was any potential correlation between the physicochemical/structural properties and the release of the drug with the in vitro permeation testing characteristics of the topical vehicle.

In spite of several studies published focusing on the evaluation of the correlation between the semi-solid vehicle and its in vitro performance when applied to the skin [18,19,20], there still remain several inconsistencies during topical product development due to the associated complexities of semi-solid products. Thus, the overall aim of the study was to conduct studies to understand and evaluate the complex nature of topically applied products, including the physicochemical and structural performance of excipients used and various physiological factors governing the permeation of drugs applied to the skin, which further contribute to several challenges in the approval of generic topical products.

## 2. Materials and Methods

### 2.1. Materials

Diclofenac sodium and benzoic acid were purchased from Sigma Aldrich (St. Louis, MO, USA). Kollisolv^®^ PEG 400 (polyethylene glycol 400), Pluriol E^®^, PEG 3350 (polyethylene glycol 3350), Kolliwax^®^ CSA 50 (cetostearyl alcohol), Kollisolv^®^, and PG (propylene glycol) were kindly provided by BASF (Tarrytown, NY, USA). Liquid paraffin was obtained from EMD Millipore (Burlington, MA, USA). Span™ and Tween™ 60 were samples from Croda Inc. (Princeton, NJ, USA), Klucel^®^ (hydroxypropyl cellulose) was provided by Ashland Inc. (Bridgewater Township, NJ, USA), and Transcutol^®^ (Diethylene glycol monoethyl ether) was provided by Gattefosse Corp. (Paramus, NJ, USA). All other reagents used were of high purity or HPLC grade.

### 2.2. Preparation of Semi-Solid Formulations

The semi-solid formulations were prepared with diclofenac sodium. Adequate pre-formulation studies were conducted in order to optimize the concentration of the different excipients based on visual changes in the appearance, microscopic evaluation, and any change in the physical characteristics prior to manufacturing the final formulations. The compositions of the final optimized formulations are shown in Table 1.

### 2.3. Manufacturing Process for Emulsion

At 70 °C, Span 60 and CSA 50 were dissolved in liquid paraffin to make the oil phase of the emulsion, and Tween 60 was dissolved in water to make the aqueous phase of the emulsion. The drug was dissolved in a mixture of PG and Transcutol and then added to the aqueous phase. The oily phase was added to the aqueous phase and homogenized at 8000 RPM using a high shear homogenizer (Omni Inc., Atlanta, GA, USA). The formulation was then cooled to room temperature while mixing continuously to form a smooth emulsion.

### 2.4. Manufacturing Process for Emulgel

The emulsion portion of the emulgel was manufactured using the same process as the emulsion. In addition, the gel portion was prepared by dispersing 1% Klucel^®^ in water at room temperature and mixing until a homogeneous dispersion was obtained. The emulsion was then mixed with the gel in a 1:1 ratio with gentle mixing to obtain the emulgel [21].

### 2.5. Manufacturing Process for Gel

The gel formulation was made by slowly dispersing Klucel^®^ in water while stirring continuously and allowing the dispersion to hydrate for 60 minutes. The drug was dissolved in a suitable amount of PG and Transcutol^®^ before being transferred to the container with the dispersion and continuously stirred until a clear homogenous gel was produced.

### 2.6. Manufacturing Process for Ointment

PEG 400 was heated to 70 °C, and the drug was then added and mixed until dissolved. PEG 3350 was then added to the mixture, and the mixture was stirred continuously and then cooled to room temperature until completely congealed.

### 2.7. Microscopic Evaluation and pH Measurement

Polarized light microscopy was used to evaluate all the formulations. A small amount of each product was placed on the glass slide and spread evenly using the coverslip. The formulations were observed under a bright-field microscope using 40× objective, and photomicrographs were recorded using the Leica DM 750 microscope (Leica Biosystems, Richmond, IL, USA).

### 2.8. pH Measurement

The pH meter (Thermo Fisher Scientific, Waltham, MA, USA) was calibrated using buffered standards of pH 4, 7, and 10. Approximately 10 g of each formulation was taken in a suitable container and tapped to remove entrapped air. The pH for all the formulations was recorded, and the probe was washed with the DI water and 70% *v/v* ethanol after each measurement.

### 2.9. Rheological Characterization

The rheological behavior of the different formulations was evaluated by using a stress-controlled rheometer (MCR-302, Anton-Paar USA Inc., Ashland, VA, USA). The equipment consisted of a step-peltier stage and a 25 mm sandblasted parallel plate. In order to keep the formulation’s temperature at 32 °C and avoid any possible evaporation, a peltier hood was used along with the fixture. All measurements were performed in triplicate. For each test, approximately 0.5 g of sample was placed on the lower plate before slowly lowering the upper plate to the preset gap of 100 microns. In order to characterize the rheological behavior, each sample was subjected to a steady-state-flow method (0.1–100 s^−1^) to characterize the flow property and to obtain viscosity values at low (2.0 s^−1^), medium (20.0 s^−1^), and high (75.0 s^−1^) shear rates. 

In addition, dynamic oscillatory tests can be used to evaluate the microstructure of a viscoelastic material. After identifying the linear viscoelastic region (LVR) by using the strain sweep for each of the individual formulations, a frequency sweep was performed over an angular frequency range of 0.1–100 rad/s in order to understand the viscoelastic nature of the tested formulations.

### 2.10. In Vitro Release Test

The IVRT method was performed using vertical diffusion cells with 5 mL volume and a recirculating water bath set at 32 ± 0.5 °C (Logan Instruments Corp., Somerset, NJ, USA).

Each diffusion cell had a donor and receptor chamber separated by a synthetic membrane. The drug product (approximately 300 mg) was applied onto the membrane in the donor chamber, and the receptor compartments were filled with 10mM phosphate-buffered saline (PBS, pH 7.4) and continuously stirred with a magnetic stirrer. The rate of drug release was determined by taking samples from the receptor compartment at specific time points up to 6 h. All samples were analyzed using HPLC.

### 2.11. In Vitro Release Test Method Validation

A brief method validation was performed in order to ensure the selected IVRT method was able to discriminate between different formulations in evaluating the drug release rate.

Validation was performed by assessing membrane inertness and the linearity, precision, sensitivity, specificity, and recovery.

#### 2.11.1. Membrane Inertness

A variety of membranes were examined (VWR, Philadelphia, PA, USA), as shown in Table 2. The receiving medium was produced with a standard diclofenac sodium solution at low (10 µg/mL) and high concentrations (120 µg/mL). The tested membranes were put together into filter cartridges that are available for purchase. To evaluate the degree of drug binding to the membrane, both standard solutions were filtered through the membrane filters and analyzed in triplicate.

#### 2.11.2. Linearity and Precision

Three IVRT runs were conducted on three different days using six diffusion cells each time in order to assess the linearity, precision, and reproducibility of the IVRT method. The slope and correlation coefficient (R^2^) for the line described by the square root of time (X axis) versus the cumulative amount released per surface area (Y axis) was calculated, and R^2^ > 0.90 was found to be acceptable. The intra- and inter-run variability were estimated for the release rates, and %RSD ≤ 15% was considered acceptable.

#### 2.11.3. Sensitivity and Specificity

Sensitivity and specificity were evaluated by investigating the rate of release of the drug from the test formulations containing 0.5% and 1.0% diclofenac, respectively. The sensitivity of the IVRT method was validated by evaluating the response of the release rate to changes in the concentration of the formulations. The IVRT method was considered to be sensitive if the mean release rate was lower for the 0.5% test formulation compared to the 1.0% formulation. The specificity of the IVRT method was characterized by evaluating the change in the release rate with the concentration of the test formulation. A linear regression model was utilized to estimate the coefficient of determination (R^2^). The IVRT method was considered to be specific if the R^2^ was greater than 0.90, confirming a specifically proportional, linear relationship.

#### 2.11.4. Recovery

The results from the three IVRT runs performed during the inter-day precision investigations were used to calculate the dose depletion and recovery. The recovery was calculated by dividing the average cumulative amount released per cm^2^ at the last point in time (t = 6 h) by the applied dose (dose amount × product strength, i.e., [0.300 g formulation] × [10 mg diclofenac/g of formulation]).

Higuchi’s approximations [22] were based on the dose depletion being ≤ 30.00% in spite of inherent differences in the physicochemical properties of the different semi-solid formulations. This means that steady-state conditions during the IVRT run would not be affected and consequently impact the linearity of the method. Hence, a recovery indicating a dose depletion of ≤30.00% was considered to be acceptable.

### 2.12. In Vitro Permeation Testing

Cryopreserved, dermatomed human skin was obtained from a skin bank (New York FireFighters, New York, NY, USA). Prior to the experiment, the skin was thawed and cut into sections large enough to mount on vertical Franz diffusion cells with a diffusion area of 0.64 cm^2^ (PermeGear, Philadelphia, PA, USA). The receptor chamber was filled with 10 mM PBS (pH 7.4), and the diffusion apparatus was temperature-controlled in order to maintain the surface skin temperature at around 32 °C. The thickness of dermatomed human skin used for IVPT studies was 400–500 µm.

The barrier integrity of the skin was evaluated by measuring the electrical resistance before the application of the formulations at a frequency of 100 Hz and a low voltage of 100 mV. Skin specimens in which the resistance was found to be acceptable were used for further permeation studies [23]. A finite dose of 10 mg/cm^2^ of the formulation was added to the donor compartment. Sink conditions were maintained throughout the experiment. After 24 h, the excess formulation was wiped off with a cotton swab. Any formulation remaining on the skin surface was also removed by tape stripping (CuDerm Corp., Dallas, TX, USA) by using up to 2 consecutive strips. The remaining stripped skin, including the epidermis and dermis, was collected separately for analysis. The cotton swabs, the tape strips, and stripped skin were placed in a vial containing 10 mM PBS pH 7.4, and the mixtures were stirred overnight in order to ensure adequate drug extraction.

### 2.13. Quantitative Analysis

The Alliance HPLC Waters 2695 Separations Module connected to a Waters UV detector was used to examine the samples. A 250 × 4.6 mm, 5 C8 Luna column was used for the HPLC assay (Phenomenex, Torrance, CA, USA). Methanol (66% *v/v*) and 10 mM sodium phosphate buffer (34% *v/v*) (adjusted to pH 3.0 with o-phosphoric acid) was the mobile phase. The wavelength for detection was 276 nm, while the flow rate and injection volume were set at 1.2 mL/min and 20 µL, respectively. A calibration curve of standards with concentrations ranging from 0.1 µg/mL to 50 µg/mL was run. The linearity between the peak area of diclofenac sodium standard solutions and their concentrations was found to be good, with a high correlation coefficient of R^2^ > 0.99.

### 2.14. Statistical Analysis

The data obtained were subjected to statistical analysis using One-way Analysis of Variance (ANOVA) following Tukey’s test. A value of *p* ≤ 0.01 was considered statistically significant.

As the aim of this research was not to determine bio(in)equivalence between the different semi-solid formulations, the number of replicates employed was not based on rigorous calculations. Additionally, the Mann–Whitney U test, which is typically used for IVRT studies, was not used in this study, as it has been historically used to compare the test and reference product [24].

## 3. Results

### 3.1. Visual Evaluation and pH Measurement of the Semi-Solid Formulations

The formulations shown in Table 1 were evaluated based on their visual appearance. The ointment was opaque, while the emulsion was smooth, white, and homogenous in appearance. The gel formulation was clear and homogenous, while the emulgel was found to be translucent and homogenous in appearance. The pH of the gel, emulgel, and emulsion was found to be in the acceptable range of 7.0–7.1.

### 3.2. Microscopic Evaluation

**Figure 1:**
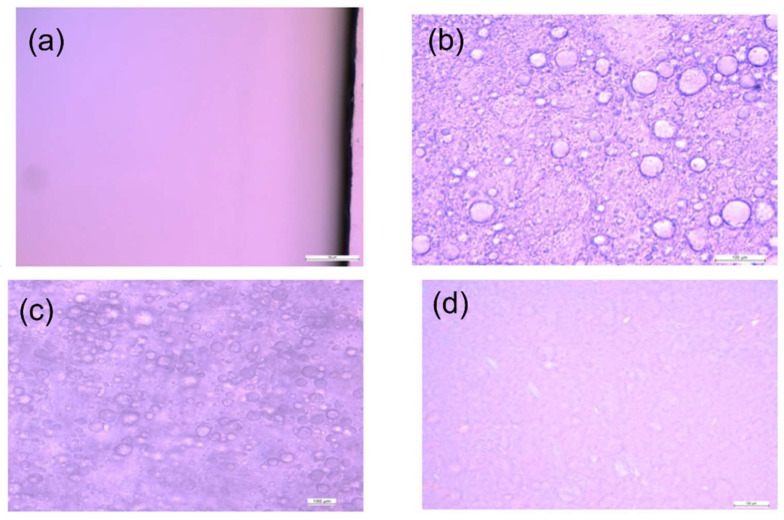
Microscopic observation of (**a**) Gel, (**b**) Emulgel, (**c**) Emulsion, and (**d**) Ointment at 400× magnification. Scale bar represents 100 µm.

All formulations were evaluated under the microscope using a 40× objective. The microscopic pictures (Figure 1) show that diclofenac is in the dissolved form, and no crystals of API are visible under the microscope for the gel and ointment formulation. In the case of the emulsion and emulgel formulation, typical globules were visible under the microscope with no drug crystals.

### 3.3. Rheological Characterization

Understanding the rheology of the formulation is important for semi-solid pharmaceutical dosage forms because it may have an impact on the way the therapeutic agent is applied and delivered. Therefore, research was conducted to determine how different processing parameters or stressors affected the rheological characteristics of the semi-solid formulations.

#### 3.3.1. Flow Curve (Viscosity vs. Shear Rate)

**Figure 2:**
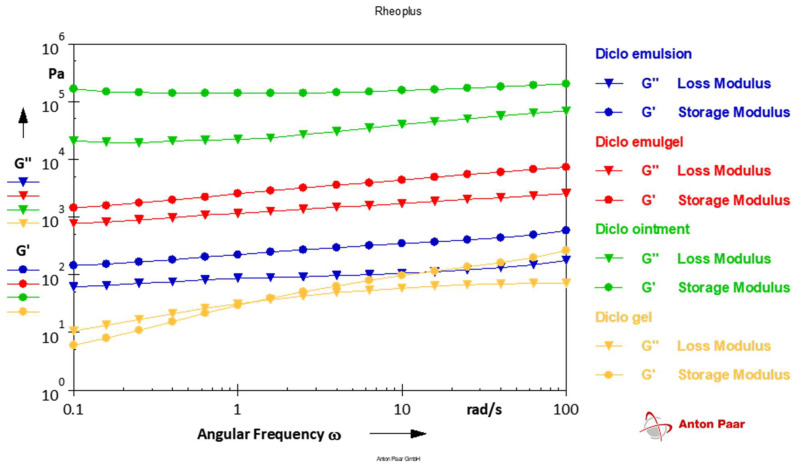
Frequency sweep test for different semi-solid formulations containing diclofenac sodium (1%).

As seen in Table 3 and Figure 2, the viscosity of the ointment was found to be the highest among all the formulations. All the semi-solid formulations were found to be characteristically shear-thinning and non-Newtonian with increasing shear rate. This means that at low shear, the formulations displayed high viscosity, which represents the initial physical stability/firmness of the product. As the shear rate was increased, the viscosity of the formulation quickly decreased, which represents the ease of spreadability on the application of the formulation topically.

#### 3.3.2. Linear Viscoelastic Response

All of the formulations exhibited similar viscoelastic behavior when subjected to increasing strain, with storage modulus (G′) greater than loss modulus (G′′). This shows that cohesive forces were heavily predominant in the microstructure of the formulations. This property allows for easy application on the skin without any potential drip-off.

### 3.4. In Vitro Release Test Method Validation

Select parameters were tested on the investigational formulations in order to confirm the ability of the method to differentiate between formulations with different compositions. The IVRT validation was performed with test formulations containing 1% and 0.5% diclofenac sodium. Membrane inertness, as well as the solubility of the drug in the receptor medium, were determined. In addition, the linearity, precision, sensitivity, specificity, and recovery of the IVRT method were also evaluated. Results are depicted below.

#### 3.4.1. Membrane Selection and Solubility of Diclofenac in the Receptor

The solubility of diclofenac sodium in PBS (pH 7.4) has been reported [25] as 6.18 ± 0.48 mg/mL, which is higher than the concentrations obtained during the method validation experiments and ensured the receiving medium provided a ‘diffusional sink’ for the release of the active ingredient from the formulation.

The filtered standard solutions were analyzed in order to evaluate drug recovery. As seen (Table 4) in the Nuclepore membrane showed the highest recovery for both the lower and higher concentration solution compared to all the other membranes, which means that the membrane was inert and offered the least resistance to the free diffusion of the drug.

Based on the membrane binding studies, the Nuclepore membrane was used to evaluate the in vitro release of the semi-solid formulations.

#### 3.4.2. Linearity, Precision, and Reproducibility

Sample solutions of the receptor medium in each cell were withdrawn at time points of 0.5, 1, 1.5, 2, 3, 4, 5, and 6 hours for analysis in order to generate a satisfactory release profile and to characterize the mechanism and type of release from the semi-solid products.

Least squares linear regression analysis was applied to determine the linearity of the IVRT profile of diclofenac sodium during the 6-hour run. The coefficient of linearity (R^2^) was greater than 0.90 in all cases, which suggests that the release of API into the receptor medium was linear with the square root of time, thus following Higuchi’s model.

**Figure 3:**
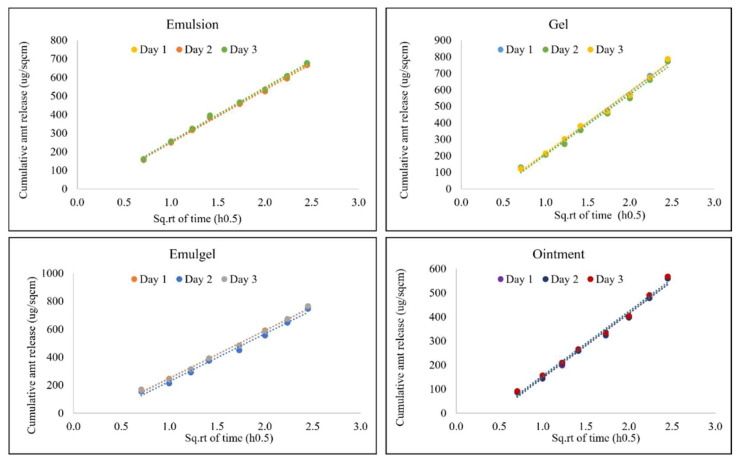
Inter-day precision for all semi-solid formulations. Data shown as mean release rate (µg/cm^2^/min^0.5^) and % RSD for six replicates.

**Figure 4:**
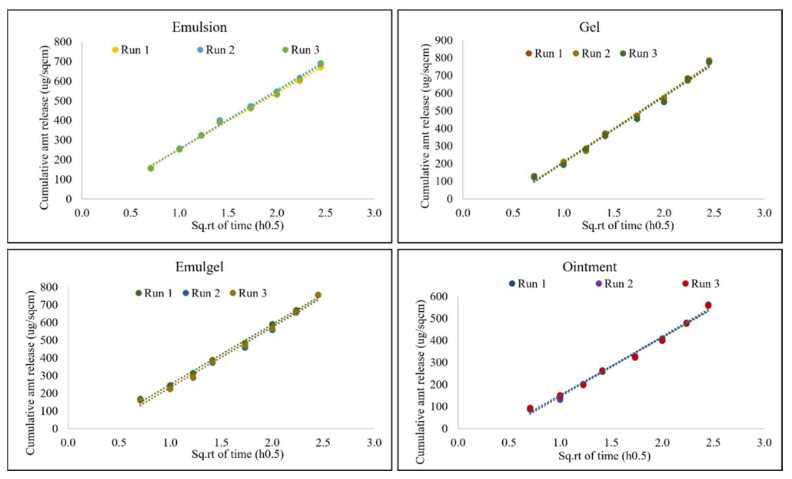
Intra-day precision for all semi-solid formulations. Data shown as mean release rate (µg/cm^2^/min^0.5^) and % RSD for six replicates.

The precision of the method was determined by performing inter-day and intra-day precision runs (Figure 3 and Figure 4). The inter-day precision was evaluated by testing the same batch for all formulations with identical compositions on three different days, as seen below. The %RSD of in vitro release rate (Slope, µg/cm^2^/min^0.5^) for all samples was found to be less than 15%. The data generated from these tests are listed in Table 5 and plotted in Figure 3 as the cumulative amount of drug released per unit area vs. square root of time.

The intra-day precision run was evaluated by testing the same batch on the same day at different times. As seen in Table 6 and Figure 4, the mean release rate values were found to have %RSD less than 15% indicating that the method was precise.

#### 3.4.3. Sensitivity, Specificity, and Recovery

IVRT method should be able to discriminate release rates from similar formulations in which the concentration of API has been altered with higher strength or lower strength. All the formulations shown in Table 1 were prepared by the same process but contained 0.5% drug. Comparative analysis of products containing 50% API was performed against 100% label claim as a reference to demonstrate the IVRT method sensitivity to concentrations.

**Figure 5:**
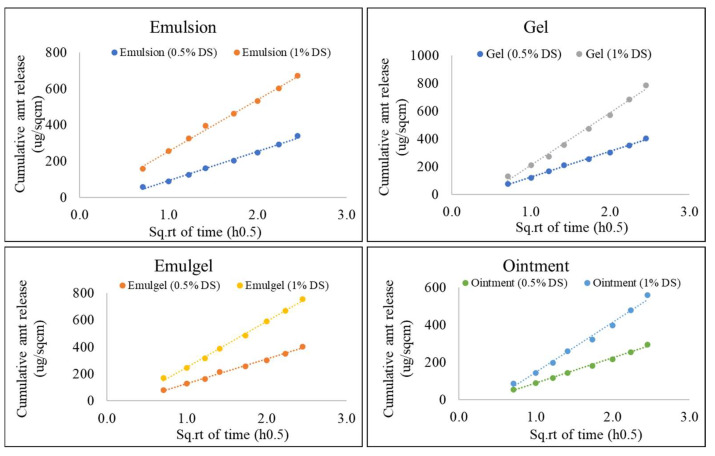
Effect of dosage strength on release rate for all formulations. Data shown are mean of six replicates.

The mean release rate and amount of drug released were found to be proportional to the dosage strength for all the formulations, as seen in Figure 5 and Table 7. Therefore, the developed IVRT method was found to be sensitive to different concentrations of diclofenac sodium in the formulation.

The specificity of the method was determined by evaluating the relationship between the changes in release rate with the strength of the formulation. The linear regression model showed that the coefficient of determination (R^2^) was greater than 0.90, indicating a specifically proportional and linear relationship.

Calculated dose depletions for the three IVRT runs are shown in Table 8. Since the dose depletion was found to be less than 30.00% and in part because of the acceptable linearity of the API release rates throughout the duration of the IVRT study, the extent of dose depletion was considered to be acceptable.

### 3.5. In Vitro Release Profile

**Figure 6:**
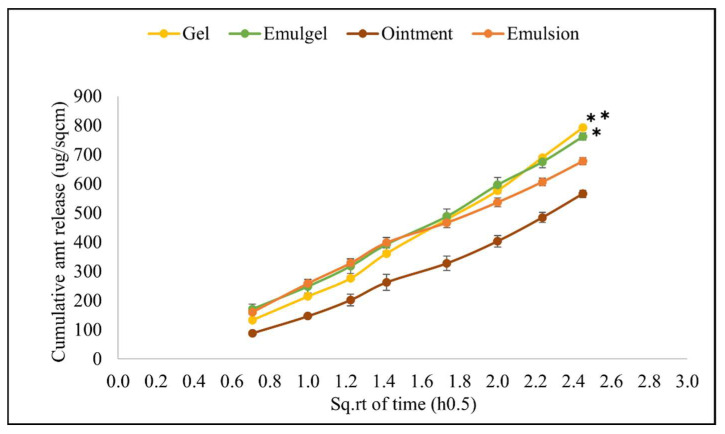
In vitro release profile of different semi-solid formulations, ** indicates *p* < 0.01 and statistically significant in comparison to all other formulations, * indicates *p* < 0.01 and statistically significant in comparison to emulsion and ointment. Data shown are Mean ± SD of six replicates.

**Figure 7:**
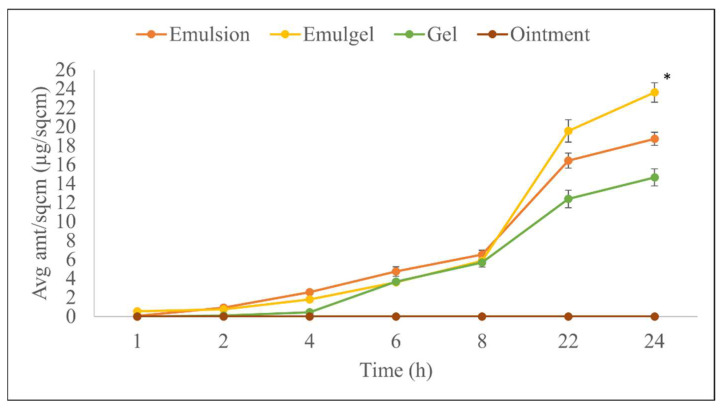
In vitro permeation comparison of different semi-solid formulations, * indicates *p* < 0.01 and statistically significant in comparison to all other formulations. Data shown are Mean ± SE of six replicates.

The validation results confirmed the suitability of the IVRT method to measure the release rate of diclofenac sodium from the semi-solid formulations. When the cumulative amount of drug diffused per cm^2^ was plotted against the square root of time, a linear relationship was observed with a correlation coefficient greater than 0.90 for all formulations. The average cumulative amount at the end of 6 h and the mean release rate were found to be significantly higher for the gel formulation (792.10 ± 3.07 μg/sq.cm and 380.42 ± 3.05 μg/sq.cm/h^0.5^, respectively) compared to all the other formulations as seen in Figure 6 and Figure 7. The emulgel showed a significantly higher cumulative release and mean release rate (762.30 ± 12.57 μg/sq.cm and 342.38 ± 5.72 μg/sq.cm/h^0.5^, respectively) than the emulsion and ointment formulations. The release rate for the emulsion formulation also differed significantly when compared to the ointment formulation [26].

### 3.6. In Vitro Permeation Test and Skin Distribution

In vitro drug permeation can detect differences in topical drug delivery when formulations of varying compositions are used. The average cumulative amount of drug permeation through human skin at the end of 24h was found to be 23.61 ± 1.03 µg/cm^2^ for the emulgel, 18.72 ± 0.69 µg/cm^2^ for the emulsion, and 14.66 ± 0.91 µg/cm^2^ for the gel, while no drug was found in the receptor for the ointment formulation. The low permeation from the ointment formulation could be correlated to its highest viscosity in comparison to all other formulations [26].

**Figure 8:**
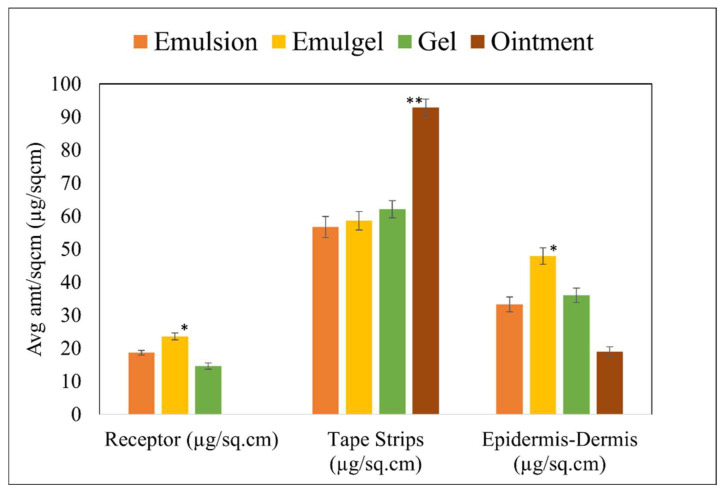
Comparison of in vitro profile using different semi-solid formulations, * and ** indicates *p* < 0.01 and statistically significant in comparison to all other formulations. Data shown are Mean ± SE of six replicates.

The functional ability of topically applied diclofenac sodium depends on drug retention in the local area as well as in the systemic circulation. As seen in Figure 8, the amount of drug retained in the tape strips for the ointment formulation was found to be significantly higher (92.90 ± 2.57 µg/cm^2^). In the case of the amount of drug in the epidermis-dermis, the emulgel formulation showed an almost 1.7-fold increase (47.95 ± 2.47 µg/cm^2^) in skin retention compared to the other semi-solid formulations.

## 4. Discussion

The physicochemical characteristics of the active ingredient, as well as the semi-solid vehicle’s composition, determine the overall efficacy of a topical formulation. NSAIDs such as diclofenac sodium (drug in our study) must first be released from the vehicle before the active ingredient may partition into the various layers of the skin. Our aim was to determine the correlation between the semi-solid vehicle and its physicochemical and structural performance and determine if there was a subsequent relationship with the in vitro performance when applied to the skin [26]. Based on this correlation, a suitable topical dosage form can be selected based on the intended therapeutic action and patient acceptance. Clinical studies have shown that in the case of a corticosteroid-containing topical formulation for the management of psoriasis, the aerosol formulation showed a greater degree of potency and tolerance compared to the ointment formulation [27]. Similarly, 15% azelaic acid gel/foam vehicles are indicated in the treatment of mild to moderate rosacea, while the 20% cream is used for topical treatment of mild-to-moderate inflammatory acne vulgaris [28]. Several studies have been published evaluating this correlation [18,19,20], but there still remain several inconsistencies during topical product development due to the associated complexities of semi-solid products. In addition, studies have shown that the microstructure of topical products is influenced by product performance which includes attributes such as pH, viscosity, in vitro drug diffusion, and skin permeation [29]. Some high-performance tests such as Confocal Raman spectroscopy and NIR spectroscopy have also been used to evaluate both the active ingredient and the effect of the topical vehicle in recent years [30,31] but still need further exploring and are beyond the scope of this research. Our findings in this study are an attempt to understand the behavior of the vehicle when applied to the skin and also why some of the excipients used in the manufacturing process were similar for the different formulations in order to minimize the effect of the excipient on the in vitro behavior of the formulation [32].

The appearance of all the semi-solid formulations used in this study was examined visually as well as under the microscope. None of the formulations showed any presence of drug crystals or phase separation during this period. The pH of the formulations was found to be acceptable, indicating that the formulations were physicochemically stable.

The rheological properties were determined at 32°C in order to simulate the spreading of these semi-solid formulations on the skin and could provide a good correlation with patient acceptance. The viscosity of the formulations was determined at low, medium, and high shear rates. Low shear represents the viscosity at resting or in its final packaging configuration, medium shear represents the viscosity during squeezing from packaging, and high shear represents the viscosity during rubbing on the skin. All the formulations exhibited a characteristic pseudoplastic shear-thinning behavior with higher viscosity at a low shear rate which quickly decreased with increasing shear. As expected, the ointment was found to be highly viscous, while the emulsion formulation showed the lowest viscosity among all the formulations.

Dynamic oscillation testing helps to understand the viscoelastic nature of the semi-solid material and can be used to predict the structural stability of the formulation [33]. In this case, the frequency sweep test showed that the G′ > G′′ indicates the predominant elastic properties of the formulations, which means that the material will return to its original structure once the deforming force has been removed. The ointment formulation showed a higher degree of elasticity, followed by the emulgel in comparison to the other formulations. A crossover of the G′ and G′′ at a relatively low strain in the instance of the gel formulation indicates that the formulation slowly becomes more fluid-like and is a sign of the relaxation of the internal structure of the network the polymer produced. This characteristic of hydroxypropyl cellulose-containing gels has been discovered, and it denotes a larger elastic contribution that can be connected to physical properties such as pourability and processability [34].

Given that the test is founded on good scientific principles and was initially used to determine product sameness between pre- and post-change topical dosage forms, IVRT has been acknowledged as an important tool in the development of topical semi-solid formulations [35,36]. IVRT tests are typically used to show Q3 similarity between the generic test and reference product which are Q1/Q2 similar, or for scale-up and post-approval changes (SUPAC). In research and development, this test can be used to predict the performance of the semi-solid formulation as the √t–release rate is a distinguishing property of each formulation and may be indicative of the clinical performance of the particular semi-solid formulation [37]. Tiffner et al. conducted a similar study where the objective was to determine if an in vitro release test (IVRT) could distinguish between the release rates of five acyclovir topical products from those of a reference product, Zovirax cream (USA). They observed that when two products are determined to have equal drug release rates using a validated IVRT approach, the likelihood that there may be clinically significant discrepancies between the products is significantly reduced [38].

This study demonstrated that the Nuclepore membrane had no evident resistance to the drug’s diffusion. The Higuchi diffusion model was found to be followed by the release of diclofenac sodium from the various vehicles, and the drug release was found to be less than 30%, satisfying the requirements of a standard IVRT [39,40]. The developed method was found to be able to distinguish between the formulations, was discovered to be able to detect changes in dosage strength, and also assured a good correlation between the uniformity of the same and different batches for all the formulations upon testing the various parameters for validation. As a result, the methodology was determined to be “valid” for all the parameters tested. The gel formulation showed the highest drug release, followed by the emulgel formulation in comparison to all the formulations, potentially due to the lower viscosity and fluid-like nature of the formulations. Previous studies have shown that formulations with higher viscosity, such as ointment, exhibit lower release for NSAID drugs such as tiaprofenic acid [41].

In vitro permeation has been used extensively as a surrogate for clinical BE studies and provides a direct evaluation of drug permeation into and across the skin [42,43]. The IVPT experiments showed that the topical delivery of diclofenac sodium was significantly higher for the emulsion and emulgel formulation. The amount of drug retained in the tape strips was found to be significantly higher for the ointment formulation, while no drug was found in the receptor, which means that a majority of the drug remained on the skin surface with minimal distribution in the epidermis-dermis. The emulgel formulation showed a higher degree of skin retention, while no significant difference was observed in the epidermal-dermal layers between the gel and emulsion formulation. In the case of the emulgel formulation, the higher skin retention and permeation may be due to the dual property of the emulsion, which is highly shear thinning and readily penetrates into the skin, and the gel, which provides improved stability and penetration ability when applied topically [44]. In addition, favorable rheological properties can also improve drug release and skin penetration for emulgel-type formulations [45].

Cordery et al. conducted a study to assess the effectiveness of stratum corneum tape-stripping in vivo and in vitro skin permeation as techniques for measuring the bioavailability of diclofenac applied topically from three approved topical products (two gels and one solution) [46]. When the topical bioavailability of marketed products containing diclofenac sodium was tested both in vitro and in vivo, a strong IVIVC was observed [46], which corroborates the use of IVPT as a complementary surrogate tool to optimize the formulation performance and generate relevant information regarding the bioavailability of topical drugs whose site of action lies below the skin barrier. An attempt was made to understand the correlation between the IVRT and IVPT results in this study, and it was observed that a direct correlation may not always be possible simply due to the complexity of the skin and the formulation. The gel formulation showed the highest release among all the formulations but comparably lesser skin permeation and retention compared to the emulsion formulation, where a lower drug release but greater delivery in the skin was observed. However, the emulgel formulation showed a relatively similar trend for both IVRT and IVPT. Similar observations have been previously reported for emulgel formulations in a study by Li et al., which focused on understanding the correlation between THP’s percutaneous penetration, rheological characteristics, and in vitro release could lead to significant insights and make it easier to create customized emulgels [45]. On the other hand, the ointment showed the lowest drug release with negligible permeation and higher cutaneous retention. This suggests that every formulation should be evaluated on a ‘case-by-case’ basis, and IVRT may be suitable only for evaluating formulations that are Q1/Q2 similar or during SUPAC changes as recommended by the FDA. However, both rheology and IVRT testing provide a means of early screening testing prior to IVPT and provide a better understanding of manufacturing variables which can then be potentially related to skin permeation.

The therapeutic effect of dermatological products depends on their ability to act locally in the epidermal/dermal layers of the skin. A single ‘gold standard’ method is not available currently to determine the bioavailability of topical formulations; hence, a combination of different techniques is required in order to assess the in vitro performance of the formulation prior to performing clinical studies, which can be expensive and provide insensitive outcomes [42]. While evaluating the performance of the different formulations used in our study, the emulgel or gel formulation may be more beneficial for the treatment of pain due to the ease of spreadability and elasticity, enhanced release, permeation, and skin retention in the epidermal-dermal layers. On the other hand, the emulsion formulation may be more conducive to treating inflammatory skin conditions due to both the emollient nature of the product as well as enhanced cutaneous retention compared to the ointment. These findings confirm that depending on the site of action and intended therapeutic effect, the selection of a suitable vehicle plays a critical role in the in vitro performance and permeation of semi-solid formulations.

## 5. Conclusions

The semi-solid formulations with diclofenac sodium used in this study were found to be stable and exhibited characteristic rheological behavior, which was found to be favorable for topical application. The IVRT method was able to differentiate changes in the composition of the vehicles, with the gel and emulgel formulations exhibiting the highest release rate among all the formulations. Based on permeation and skin distribution studies, the emulgel formulation showed favorable skin permeation and retention, while the gel and emulsion were more conducive to skin retention indicating the effect of the formulation on their in vitro performance. Although the clinical relevance of these differences in the performance of different formulations is unknown, it does highlight the importance of Q1/Q2 similarity for a generic product. The authors also acknowledge that the results are specific to the formulations developed and can vary with a range of topical excipients that can be used for developing these formulations. Each drug has different physicochemical properties, so the results of this study may not be broadly applicable to other drugs. The results showcase the use of these tests as a pre-formulation tool for the development of topical formulations with good aesthetic acceptability and efficacy.

## Figures and Tables

**Table 1:** Composition of different semi-solid formulations (% *w/w*).

Components	Emulsion (% *w/w*)	Emulgel (% *w/w*)	Gel (% *w/w*)	Ointment (% *w/w*)
**Diclofenac sodium**	1.00	1.00	1.00	1.00
**Propylene glycol**	5.00	5.00	5.00	-
**Transcutol^®^**	10.00	10.00	10.00	-
**Ceto Stearyl Alcohol 50**	2.00	2.00	-	-
**Liquid Paraffin**	7.50	7.50	-	-
**Span™** **60**	4.50	4.50	-	-
**Tween™ 60**	0.50	0.50	-	-
**Klucel^®^**	-	1.00	2.00	-
**PEG 400**	-	-	-	59.00
**PEG 3350**	-	-	-	40.00
**Benzoic acid**	0.25	0.25	0.25	-
**Water**	Q.s. to 100%	Q.s. to 100%	Q.s. to 100%	-
**TOTAL**	100%			

**Table 2:** Commercially available membranes and their dimensions.

Membrane	Pore Size	Diameter
Cellulose acetate (OE 67)	0.45 µ	25 mm
Nuclepore Track-etched Polycarbonate	0.4 µ	25 mm
HT Tuffryn Polysulfone	0.45 µ	25 mm
Nylon	0.45 µ	25 mm

**Table 3:** Low, Medium, and High Shear Viscosity Values for the semi-solid formulations containing diclofenac sodium (1%).

Shear Rate (s^−1^)		Viscosity (cP)			
		Gel	Emulgel	Emulsion	Ointment
**Low shear**	**2.0**	52,367	39,167	10,633	908,667
**Medium shear**	**20.0**	10,997	3947	885	101,467
**High shear**	**75.0**	4053	1353	323	27,067

**Table 4:** Drug recovery from standard solutions after passing through different membranes.

Membrane	Conc. (µg/mL)	Drug Recovery ± SD (%) (n = 3)
**Cellulose acetate**	10	88.95 ± 0.20
	120	96.57 ± 0.66
**Nylon**	10	50.19 ± 0.69
	120	29.00 ± 4.35
**Tuffryn**	10	64.13 ± 0.93
	120	97.97 ± 1.87
**Nuclepore**	10	95.78 ± 0.07
	120	99.49 ± 1.07

**Table 5:** Inter-day precision data for the semi-solid formulations. Data shown as mean release rate (µg/cm^2^/min^0.5^) and % RSD for six replicates.

Formulation	Mean Release Rate on Day 1 (µg/cm^2^/min^0.5^)	Mean Release Rate on Day 2 (µg/cm^2^/min^0.5^)	Mean Release Rate on Day 3 (µg/cm^2^/min^0.5^)	Mean Release Rate (µg/cm^2^/min^0.5^)	% RSD
**Emulsion**	284.84	284.87	289.14	286.28	0.86
**Gel**	378.21	366.06	372.42	372.23	0.66
**Emulgel**	340.17	340.56	345.33	342.02	0.84
**Ointment**	268.22	265.87	268.29	267.46	0.52

**Table 6:** Intra-day precision data for the semi-solid formulations. Data shown as mean release rate (µg/cm^2^/min^0.5^) and % RSD for six replicates.

	Mean Release Rate for Run 1 (µg/cm^2^/min^0.5^)	Mean Release Rate for Run 2 (µg/cm^2^/min^0.5^)	Mean Release Rate for Run 3 (µg/cm^2^/min^0.5^)	Mean Release Rate (µg/cm^2^/min^0.5^)	% RSD
**Emulsion**	284.84	298.83	295.92	293.20	2.52
**Gel**	378.21	375.25	373.49	375.65	0.63
**Emulgel**	340.17	340.97	346.18	342.44	0.94
**Ointment**	268.22	272.33	262.50	267.68	1.84

**Table 7:** Comparison of mean release rate (slope) values for different dosage strengths of semi-solid formulations containing diclofenac sodium.

Formulations	Mean Slopes for 0.5% Strength Formulations	Mean Slopes for 1% Strength Formulations
Emulsion	160.86	284.84
Gel	185.58	378.21
Emulgel	181.73	340.17
Ointment	135.01	268.22

**Table 8:** Percentage dose depletions for all semi-solid formulations from three IVRT runs.

Formulations	Dose Depletions ± SD (%) from Precision Runs (n = 3)
Emulsion	14.33 ± 0.13
Gel	16.68 ± 0.17
Emulgel	16.12 ± 0.20
Ointment	11.99 ± 0.10

## Data Availability

Data is contained within the article.
